# Cardiovascular disease is increased prior to onset of rheumatoid arthritis but not osteoarthritis: the population-based Nord-Trøndelag health study (HUNT)

**DOI:** 10.1186/ar4527

**Published:** 2014-04-02

**Authors:** Helen Pahau, Matthew A Brown, Sanjoy Paul, Ranjeny Thomas, Vibeke Videm

**Affiliations:** 1 University of Queensland Diamantina Institute, Translational Research Institute, Princess Alexandra Hospital, Woolloongabba, QLD, Australia; 2 Queensland Clinical Trials and Biostatistics Centre, School of Population Health, University of Queensland, Princess Alexandra Hospital, Wooloongabba, QLD, Australia; 3 Department of Laboratory Medicine, Children’s and Women’s Health, Norwegian University of Science and Technology, Trondheim, Norway; 4 Department of Immunology and Transfusion Medicine, Trondheim University Hospital, Trondheim, Norway; 5 Current address Clinical Trials & Biostatistics Unit, QIMR Berghofer Medical Research Institute, Herston, QLD, Australia

## Abstract

**Introduction:**

Patients with rheumatoid arthritis (RA) have increased risk of cardiovascular (CV) events. We sought to test the hypothesis that due to increased inflammation, CV disease and risk factors are associated with increased risk of future RA development.

**Methods:**

The population-based Nord-Trøndelag health surveys (HUNT) were conducted among the entire adult population of Nord-Trøndelag, Norway. All inhabitants 20 years or older were invited, and information was collected through comprehensive questionnaires, a clinical examination, and blood samples. In a cohort design, data from HUNT2 (1995–1997, baseline) and HUNT3 (2006–2008, follow-up) were obtained to study participants with RA (*n* = 786) or osteoarthritis (*n* = 3,586) at HUNT3 alone, in comparison with individuals without RA or osteoarthritis at both times (*n* = 33,567).

**Results:**

Female gender, age, smoking, body mass index, and history of previous CV disease were associated with self-reported incident RA (previous CV disease: odds ratio 1.52 (95% confidence interval 1.11-2.07). The findings regarding previous CV disease were confirmed in sensitivity analyses excluding participants with psoriasis (odds ratio (OR) 1.70 (1.23-2.36)) or restricting the analysis to cases with a hospital diagnosis of RA (OR 1.90 (1.10-3.27)) or carriers of the shared epitope (OR 1.76 (1.13-2.74)). History of previous CV disease was not associated with increased risk of osteoarthritis (OR 1.04 (0.86-1.27)).

**Conclusion:**

A history of previous CV disease was associated with increased risk of incident RA but not osteoarthritis.

## Introduction

Rheumatoid arthritis (RA) is a systemic, inflammatory autoimmune disorder that primarily involves the joints. Despite treatment advances, RA patients continue to have higher mortality and morbidity rates than the general population, predominantly related to increased atherosclerotic cardiovascular (CV) disease [1,2]. The traditional CV risk factors include age, gender, family history, smoking, diabetes, hypertension, dyslipidemia, obesity and sedentary lifestyle. RA patients carry a higher burden of some of these factors. The disease occurs most commonly after 40 years of age and is associated with accelerated cellular ageing [3]. A history of smoking is associated both with RA development and severity [4-6]. Physical inactivity is common in patients with RA and may increase co-morbidity due to obesity or diabetes, leading to an increased risk of CV mortality [7]. Some studies have also suggested that myocardial disease without atherosclerosis is more prominent in RA patients. For example, an echocardiography study demonstrated that RA patients asymptomatic for CV disease had ischemia more frequently than controls, but they also had fewer significant angiographic coronary artery stenoses [8]. Likewise, an autopsy study showed that RA patients had diffuse myocardial fibrosis or non-specific myocardial degeneration more frequently than controls after excluding individuals with other known causes [9].

Inflammation plays a central role in the pathogenesis of atherosclerosis, and the increase in CV disease and mortality in RA may partly be explained by inflammatory factors associated with RA, even after adjustment for traditional CV risk factors [10,11]. We and others showed that atherosclerosis is increased both in patients with established RA and at presentation in patients with recent-onset RA, as determined by increased carotid *intima media* thickness and plaque, and is associated with their inflammatory burden [12,13]. The risk for myocardial infarction (MI) and possible CV death is already increased within approximately 5 years after diagnosis of RA depending upon age and presence of CV risk factors, resulting in a 10-year absolute risk comparable to non-RA individuals who were 5 to 10 years older [14].

It has been demonstrated that inflammation pre-dates the onset of RA [15]. These data suggest that an increased risk of CV disease might also precede the onset of RA. The Rochester Epidemiology Project study demonstrated that RA patients were more likely to have been hospitalized because of MI prior to RA diagnosis [16]. However, a previous longitudinal cohort study found no difference in the rate of MI, congestive heart failure or angina between pre-RA and control individuals [17].

We hypothesized that CV risk factors and events are more prominent in persons with incident RA, and that this augments the risk of future RA development by increasing inflammation. The aim of the study was therefore to investigate the effects of CV risk factors and CV events on the development of RA, using a population-based cohort study design. As a control to study whether any findings were specific to RA or related to arthritis in general, a parallel investigation was performed in participants with and without osteoarthritis.

## Methods

The study participants were from the Nord-Trøndelag Health Study (HUNT) population-based health surveys conducted in the county of Nord-Trøndelag in Norway. The county is fairly representative for Norway as a whole, with a stable and ethnically homogenous population (3% non-Caucasians). All inhabitants 20 years or older were invited, and information was collected through comprehensive questionnaires and a clinical examination. The HUNT2 survey has previously been described in detail [18]. The HUNT3 survey had a similar design. In total, about 75,000 (70% of those invited) participated in HUNT2 (1995 to 1997), 51,000 (54% of those invited) participated in HUNT3 (2006 to 2008), and 37,071 participated in both HUNT2 and HUNT3. By design, the participants were not seen during the years between inclusion in HUNT2 and HUNT3.

The study cohort consisted of all participants in both HUNT2 and HUNT3 who answered whether they had a diagnosis of RA or not (n = 36,493, that is, 98.4% of 37,071) and this number determined the study size. Incident cases of RA were identified, that is, participants who reported a diagnosis of RA in HUNT3 but not in HUNT2. We also identified the incident cases of osteoarthritis for comparison, based on the question: “Has a doctor ever said that you have/have had any of these diseases: degenerative joint disease (osteoarthritis)?” The question also included the Norwegian colloquial term for osteoarthritis. Each patient group was compared to the remaining participants of the study cohort.

Participants of HUNT gave informed consent. Approval for the study was obtained from the Regional Committee on Medical Research Ethics, Central Norway, the Norwegian Data Safety Authorities and the Norwegian Department of Health. Ethics approval was also obtained by the Metro South Ethics Committee Brisbane. Permission was granted from the two primary hospitals in Nord-Trøndelag, Levanger and Namsos hospitals, and the nearest secondary referral hospital, Trondheim University Hospital, to link the identified RA cases in our study to the hospital diagnosis registries for verification of diagnosis. The registered diagnoses are used for billing and reimbursement from the national insurance scheme. We searched for the ICD-9 code 714 and ICD-10 codes M05 and M06 with sub-codes. We did not have permission to access to the patients’ case notes in order to check that the current criteria for RA were correctly employed.

On enrolment in HUNT2 and HUNT3 the participants completed a questionnaire incorporating information on medical history, smoking habits and family history of CV disease, defined as a parent, sibling or child with previous MI or stroke. Information on the use of lipid-lowering medications or non-steroid anti-inflammatory drugs was not available. Anthropometric and clinical measures included height, weight, waist and hip circumference, and blood pressure. Non-fasting blood samples were drawn and total cholesterol, low-density lipoprotein (LDL) cholesterol, high density lipoprotein (HDL) cholesterol, triglycerides and serum glucose were measured using an autoanalyzer (Hitachi Biocore Systems, Thornhill, ON, Canada). Using DNA isolated at HUNT Biobank, participants with self-reported RA were genotyped for the Shared Epitope [19]. Samples were genotyped using the Illumina Immunochip microarray chip, and HLA-DRB1 genotypes then determined by imputation using the program HLA-IMP [20,21]. Autoantibodies (anti-citrullinated peptide antibodies or ACPA, rheumatoid factor) and C-reactive protein were not measured in this survey, and classification into seropositive or seronegative RA was not possible. Hypertension was defined as systolic blood pressure ≥140 mmHg, diastolic blood pressure ≥90 mmHg or use of medication. Hypercholesterolemia was defined as total serum cholesterol >6.2 mmol/L. Body mass index (BMI) was calculated as weight/height^2^ (kg/m^2^). Patients who reported using over-the-counter analgesics daily for one month or more during the last year before inclusion in HUNT2 were recorded as users of analgesics. Previous CV disease was defined as a composite of angina, MI or stroke. The relevant questions were; “Have you had or do you have angina pectoris?” “Have you had a stroke/brain hemorrhage?” and “Have you had a myocardial infarction?” The question about angina also included a Norwegian colloquial term for this diagnosis. The composite variable was used for previous CV disease as numbers of cases were too low for analysis of individual disease events. The level of missingness at baseline for most key variables was <1%, with the exception of smoking (12.8% missing data). Missingness for smoking was evenly distributed among the subgroups of the study cohort. Non-complete cases were omitted from analysis.

### Statistical methods

Data are presented as number (percentage), mean (standard deviation) or median (interquartile range), as appropriate. The chi-square test and the Mann-Whitney U-test were used for between-group comparisons of categorical and continuous study parameters, respectively. Risk factors associated with the development of RA and osteoarthritis between HUNT2 and HUNT3 were identified using multivariate logistic regression models. Linearity of logits for continuous variables was checked by plotting. *P*-values <0.05 were considered significant. Pearson’s correlation coefficient R was calculated to evaluate linear correlation.

Since this was a population-based survey and RA was self-reported, we performed several sensitivity analyses to support our main analysis. Because previous studies have indicated that few women with RA surveyed in the community report their diagnosis accurately [22], we repeated the analysis in several subgroups of the incident RA cases using additional criteria to remove false-positive diagnoses, that is, 1) excluding all who also reported having psoriasis (n = 178), 2) including only patients where the diagnostic registries of the nearest hospitals showed a diagnosis of RA (n = 216), 3) restricting the hospital-diagnosed cases to those where the diagnosis first occurred after 1999 or 4) after 2001, to avoid including patients who forgot to report RA in HUNT2, or 5) including only incident RA cases who carried the shared epitope (with and without additional exclusion due to a report of psoriasis). To represent the CV risk factors in another way, we developed alternative models including the Framingham risk score, which is based on age, serum cholesterol, hypertension, smoking and diabetes [23], as well as gender and history of CV disease. The Framingham risk score was preferred because it assigns higher risk with diabetes and there are no age limits, and because it may easily be calculated in large cohorts. The European HeartScore [24] is based on risk for patients between 40 and 65 years and requires separate input for each individual using charts or an online calculator, which was not practical in our large cohort. To verify that the Framingham risk score was applicable in our cohort, we calculated the PC-based HeartScore for 50 randomly selected incident RA cases and 50 controls. Parallel Framingham risk scores and HeartScores were used to calculate an equation permitting estimation of the HeartScore in the entire cohort from their Framingham risk scores. An alternative logistic model was developed substituting the Framingham risk score with these estimated HeartScores. In addition, we compared factors associated with incident cases of self-reported RA with incident cases of self-reported osteoarthritis.

## Results

The baseline characteristics of the participants are presented in Table 1. At baseline (1995 to 1997) 33,567 participants reported not having RA, and of this group 786 (2.34%) reported RA at follow-up (2006 to 2008). This corresponds to an average annual incidence of 0.21% in women and 0.16% in men, respectively. If restricted to participants with cases identified in the local hospital registries (n = 216), the annual incidence was 0.06% in women and 0.04% in men. As expected, incident cases of RA were older and more of them were current or previous smokers and had hypertension at baseline. Incident cases of RA had significantly elevated metabolic risk factors including blood pressure, BMI, serum cholesterol and triglyceride. The estimated median (interquartile range) Framingham risk scores at baseline were 11 (7, 16) and 8 (4, 13), respectively, in incident cases and those who did not develop RA. Notably, a higher proportion of incident RA cases had a history of CV disease at baseline.

**Table 1:** Characteristics of individuals without rheumatoid arthritis (RA) or osteoarthritis (OA) at baseline

	**All participants without RA/OA**	**Later developed RA**	**Did not develop RA**	***P*-value**	**Later developed OA**	**Did not develop OA**	***P*-value**
**n**	33,567	786	32,781		3,586	29,981	
**Age**^**1**^**(years)**	46 (13)	51 (13)	46 (13)	**	52 (10)	45 (13)	**
**Sex (female)**	18,207 (54.2%)	488 (62.1%)	17719 (54.1%)	**	2,406 (67.1%)	15,801(52.7%)	**
**Smoking**				**			**
**Current**	8,901 (26.5%)	256 (32.6%)	8,645 (26.4%)		1,015 (28.3%)	7,886 (26.3%)	
**Former smoker**	7,908 (23.6%)	212 (27.0%)	7,696 (23.5%)		945 (26.4%)	6,963 (23.2%)	
**Never smoker**	15,017 (44.7%)	281 (35.6%)	14,736 (45.0%)		1,425 (39.7%)	13,592 (45.3%)	
**Hypertension**	12,186 (36.3%)	339 (43.1%)	11,847 (36.1%)	**	1,547 (43.1%)	10,639 (35.5%)	**
**Diabetes**	454 (1.4%)	19 (2.4%)	435 (1.3%)	*	61 (1.7%)	393 (1.3%)	
**Previous CV disease**	1,060 (3.2%)	52 (6.6%)	1,008 (3.1%)	**	158 (4.4%)	902 (3.0%)	**
**Angina**	673 (2.0%)	35 (4.5%)	638 (1.9%)	**	107 (3.0%)	566 (1.9%)	**
**MI**	441 (1.3%)	16 (2.0%)	425(1.3%)		49 (1.4%)	392 (1.3%)	
**Stroke**	223 (0.7%)	9 (1.1%)	214 (0.7%)		32 (0.9%)	191 (0.6%)	
**Family risk of CV disease**	15,692 (46.7%)	414 (52.7%)	15,278 (46.6%)	**	2,017 (56.2%)	13,675 (45.6%)	**
**Framingham risk score**^**2**^	9 (4 to 13)	11 (7 to 16)	8 (4 to 13)		11 (7 to 15)	8 (3 to 13)	
**Weight**^**1**^**(kg)**	76 (14)	77 (14)	76 (14)		77 (13)	76 (14)	
**BMI**^**2**^**(kg/m**^**2**^**)**	25.6 (23.5 to 28.1)	26.3 (24.3 to 29.0)	25.6 (23.4 to 28.1)	**	26.4 (24.2 to 29.0)	25.4 (23.3 to 27.8)	**
**Waist circumference**^**1**^**(cm)**	85 (11)	86 (11)	85 (11)	*	86 (11)	85 (11)	**
**Hip circumference**^**1**^**(cm)**	102 (8)	103 (8)	102 (8)	**	103 (8)	101 (6)	**
**Waist/hip ratio**^**1**^	0.84 (0.08)	0.84 (0.08)	0.84 (0.08)		0.83 (0.07)	0.84 (0.08)	**
**Systolic blood pressure**^**2**^**(mmHg)**	131 (120 to 144)	133 (122 to 146)	131 (120 to 144)	*	133 (122 to 147)	131 (120 to 143)	**
**Diastolic blood pressure**^**1**^**(mmHg)**	79 (11)	80 (11)	79 (11)	*	81 (11)	79 (11)	**
**Serum cholesterol**^**1**^**(mmol/L)**	5.8 (1.2)	6.0 (1.2)	5.8 (1.2)	**	6.1 (1.2)	5.8 (1.2)	**
**HDL cholesterol**^**1**^**(mmol/L)**	1.40 (0.39)	1.37 (0.39)	1.40 (0.39)		1.44 (0.40)	1.39 (0.38)	**
**Serum triglycerides**^**2**^**(mmol/L)**	1.40 (0.98 to 2.07)	1.51 (1.10 to 2.24)	1.40 (0.97 to 2.06)	**	1.46 (1.02 to 2.12)	1.37 (0.95 to 2.04)	**

^1^Values are mean (standard deviation) or numbers (%), or ^2^median (interquartile range). Non-respondents were excluded. BMI*,* body mass index; CVD*,* cardiovascular disease; HDL*,* high density lipoprotein; MI, myocardial infarction; OA, osteoarthritis; RA, rheumatoid arthritis. ^*^P-value <0.05, ^**^P-value <0.001, comparing individuals who developed RA and did not develop RA, or comparing individuals who developed OA and did not develop OA. All blood samples were non-fasting.

Female gender, age, smoking, BMI and previous CV disease were more prominent in those developing RA (odds ratio 1.52 (1.11 to 2.07), Table 2, I - Main model). There was minimal change in the odds ratio for previous CV disease with inclusion of use of analgesics in this logistic regression model, when systolic and diastolic blood pressure were included as continuous variables instead of the categorical variable for hypertension, with additional adjustment for total cholesterol and HDL-cholesterol concentrations, or when a variable for physical activity (low, moderate, high) was also included (data not shown). In all the alternative multivariate models for incident RA where the number of patients was restricted to better characterized subgroups, previous CV disease was significant and the odds ratios were higher than for the main model including all self-reported cases (Table 2, II – Alternative models A to F). A diagnosis of RA was confirmed in the hospital registries for 216 participants (27%) who had self-reported a new RA diagnosis in HUNT3. The characteristics of these participants were very similar to those reported for the entire group of incident RA cases (data not shown). In the 216 cases with a hospital diagnosis of RA, the diagnosis was found one to two years following enrolment in HUNT2 for 14 cases (6.5%), after three years for 24 cases (11.1%), after four years for 15 cases (6.9%), after five years for 28 cases (13.0%) and after six years for 22 cases (10.2%). For the remaining 113 cases (52.3%), the times were distributed from seven years onwards to the inclusion in HUNT3 with numbers varying unsystematically from 15 to 24 cases (6.9 to 11.1%) per year. Thus, there was no obvious pattern regarding the number of years from HUNT2.

**Table 2:** Effects of risk factors on incident RA and osteoarthritis, by multivariate logistic regression

	**Odds ratio**	**95% confidence interval**	***P*-value**
**I - Main model – Rheumatoid arthritis** (n = 739 patients and 30,829 controls)^1^			
Female gender	1.50	1.29 to 1.75	<0.01
Age^2^	1.03	1.02 to 1.04	<0.001
Current smoker	1.64	1.38 to 1.96	<0.001
Former smoker	1.32	1.10 to 1.59	<0.01
Hypertension	0.97	0.82 to 1.15	0.76
Diabetes	1.34	0.83 to 2.18	0.23
Body mass index^2^	1.04	1.02 to 1.06	<0.001
Previous CV disease	1.52	1.11 to 2.07	<0.01
**II - Alternative models (sensitivity analyses) - Rheumatoid arthritis**			
** A – After exclusion of patients with self-reported psoriasis** (n = 573 patients and 30,829 controls)^1^			
Previous CV disease^3^	1.70	1.23 to 2.36	0.001
** B – Including patients with hospital diagnosis of RA** (n = 201 patients and 30,829 controls)^1^			
Previous CV disease^3^	1.90	1.10 to 3.27	0.02
** C – Including patients with hospital diagnosis of RA after 1999** (n = 178 patients and 30,829 controls)^1^			
Previous CV disease^3^		2.26	1.26 to 3.99
** D – Including patients with hospital diagnosis of RA after 2001** (n = 138 patients and 30,829 controls)^1^			
Previous CV disease^3^	2.51	1.33 to 4.73	<0.01
** E – Including patients carrying the Shared Epitope** (n = 313 patients and 30,829 controls)^1^			
Previous CV disease^3^	1.76	1.13 to 2.74	0.01
** F – Including patients carrying the Shared Epitope and excluding patients with self-reported psoriasis** (n = 257 patients and 30,829 controls)^1^			
Previous CV disease^3^	1.96	1.24 to 3.10	<0.01
**III - Model for osteoarthritis** (n = 3,364 patients and 24,631 controls)^1^			
Female gender	2.36	2.15 to 2.56	<0.001
Age^2^	1.05	1.05 to 1.06	<0.001
Current smoker	1.42	1.30 to 1.56	<0.001
Former smoker	1.23	1.12 to 1.35	<0.001
Diabetes	0.90	0.66 to 1.22	0.49
Body mass index^2^	1.06	1.05 to 1.07	<0.001
Previous CV disease	1.04	0.86 to 1.27	0.66

^1^Cases with complete data.

^2^Continuous variable, Odds ratio per one unit change.

^3^Multivariate model including the same variables as the main model for RA. CV, cardiovascular; RA, rheumatoid arthritis.

In a separate alternative multivariate logistic regression model containing gender, previous CV disease and the Framingham risk score as covariates, the odds ratio for developing RA in subjects with previous CV disease was 1.65 (1.21 to 2.24) (*P* <0.01). This model also suggested the likelihood of developing RA was 6% greater with each one unit increase in Framingham risk score (odds ratio: 1.06, 95% CI: 1.05 to 1.08), *P* <0.001). The model did not change with adjustment for BMI or daily use of over-the-counter analgesics. In the model where the Framingham risk score was substituted with the estimated HeartScore, previous cardiovascular disease remained significant (odds ratio: 1.70 (1.25 to 2.32), *P* <0.01). In the 100 participants where the HeartScore was calculated using the PC-based calculator, the HeartScore (logarithmically transformed) was highly correlated with the Framingham risk score (R = 0.86, *P* <0.001). The correlation was very similar in patients (R = 0.89) and controls (R = 0.86).

At follow-up (2006 to 2008), 3,586 participants had developed osteoarthritis, corresponding to an average annual incidence of 1.05% in women and 0.64% in men, respectively. These patients were older and the proportion of smokers, former smokers and of those with hypertension was higher than in the group that did not develop osteoarthritis. Systolic and diastolic blood pressure, BMI, hip and waist circumference, total cholesterol and triglycerides were higher for incident cases of osteoarthritis, whereas HDL cholesterol was lower in incident cases of osteoarthritis. Female gender, age and smoking were associated with development of osteoarthritis, whereas previous CV disease was not (Table 2 III).

## Discussion

In the present large population-based health study we found that participants who developed RA between HUNT2 and HUNT3 had more CV disease and CV risk factors prior to the onset of RA (at HUNT2) compared to those without incident RA. A history of CV disease was a significant risk factor for incident RA, as well as the previously reported risk factors: female gender, increasing age, increasing BMI and smoking [25]. The finding of a positive association with the Framingham risk score may be related to smoking and age being incorporated into the score. The supplementary analysis showed that the Framingham score was equivalent to the estimated HeartScore in our population even though the Framingham score was not developed from European subjects. On the other hand, previous CV disease events did not contribute to the risk of future osteoarthritis in our study, indicating that the finding may be specific to RA. Smoking was associated with incident osteoarthritis.

### CV disease and incident RA

The design of our study has several strengths. Since the data on the CV risk factors and events were collected at HUNT2, recall bias at HUNT3 was greatly reduced compared to a design where patients report on these factors when receiving an RA diagnosis at a hospital-based clinic. Furthermore, the observation time between HUNT2 and HUNT3 was approximately 10 years. The importance of a long observation time is underscored by the finding that the odds ratio for the association of incident RA with previous CV disease increased when patients receiving a hospital diagnosis of RA during the first years following HUNT2 were excluded. It also seems biologically plausible that longer exposure to increased inflammation due to CV disease further increases the risk of incident RA. Furthermore, the statistical power is greatly improved in a population-based cohort study due to the large number of controls, and problems defining a relevant control group as seen with case-control studies of RA are avoided.

The major limitation of our study is that data were self-reported. Previous studies suggest that self-reported CV diagnosis and risk factors, such as hypertension and cigarette smoking, are reliable [26,27]. However, the high incidence rate of self-reported RA in our study compared, for example, to recent Swedish data based on inpatient and non-primary outpatient care (0.056% in women, 0.025% in men) [28], confirms that there probably were many false-positive RA diagnoses. This would tend to decrease the power to detect true positive results in our study, but not bias towards false-positive findings. When including only cases identified in the hospital registries, the incidence was close to the Swedish data. Furthermore, the incidence of RA increases with observation time [29]. Therefore, different data may not be directly comparable.

Epidemiological studies also carry a risk of reverse causation, which could ensue if persons with undiagnosed RA in HUNT2 were erroneously included as incident cases of RA, because RA in itself increases the risk of CV disease and events [10]. It seems unlikely that reverse causation could explain our findings, given that the association with previous CV disease became stronger when the cases with shortest duration between HUNT2 and a hospital diagnosis of RA were removed from the analysis. Furthermore, we did not observe a tendency for a higher number of cases with a hospital diagnosis to occur during the earliest years following HUNT2, which would have been expected with substantial reverse causation.

While fatal CV events prior to the onset of RA also limited the participants in our study to survivors of those events, this again would lead to under-estimation of the impact of CV events on development of RA, or to restrict the population at risk of RA to those ageing with CV risk that was insufficient to lead to premature mortality. Autoantibodies were not measured and seropositive and seronegative cases of RA could not be distinguished, which impacts on the generalizability of our results compared to others, such as population-based cohorts where it was concluded that ischemic heart disease was not increased prior to RA onset [17]. There may also have been a selection bias regarding participation in HUNT, where sicker people or those with a lower socioeconomic status are less likely to join. These people have a higher risk both for CV disease and for RA, which may have biased our findings.

The results from our various sensitivity analyses unanimously supported our main analysis. The overall conclusion that a history of CV disease is associated with the risk for incident RA therefore seems reliable. Although our study may not be suitable to give an exact estimate of the size of this increased risk, the main analysis giving an OR of approximately 1.5 is probably conservative.

### Risk factors for osteoarthritis

Our results identifying smoking as a risk factor for future osteoarthritis contradict some previous studies, which report an inverse association between smoking and osteoarthritis [30]. However, the longitudinal prospective Clearwater study designed to identify risk factors for OA development, which included 2,505 participants, did not support this inverse association with smoking for any of four joint sites (knee, hand, foot or spine) [31]. In another prospective study of 1,003 participants from the general population there was no association between smoking and radiologically-confirmed OA at different sites [32]. The current study may have shown an association with smoking because it is larger and included a greater number of incident osteoarthritis diagnoses. Nevertheless, since our study relies on self-report, replication in a cohort ascertained for radiographic osteoarthritis and symptoms is needed. However, in support of the accuracy of our case classification, we noted that higher BMI increased the risk for osteoarthritis development, as previously described [33].

### Inflammation as a link between CV disease and incident RA

From a biological perspective based on the current studies, we hypothesize that inflammation may contribute to the parallel development of atherosclerosis and RA during the pre-clinical period in individuals exposed to common RA and CV risk factors, just as this interaction accelerates complication of RA by CV events after onset.

Inflammation is the most plausible link between previous CV disease or BMI and increased risk for RA. It is well established that the pathogenesis of CV disease, that is, atherogenesis, includes chronic inflammation of the wall of muscular arteries and that inflammation is increased during acute coronary events, associated with unstable plaque and thrombosis [16]. Furthermore, obesity induces chronic inflammation in adipose tissue, increasing pro-inflammatory cytokines including interleukin-6 and tumour-necrosis factor [34]. In addition, a pro-inflammatory state is present before the clinical onset of RA. C reactive protein (CRP) levels were found to be higher in individuals with pre-clinical RA compared to a control group [35]. Serum cytokines and chemokines were also elevated preceding the onset of RA [36]. In at least some patients, periodontal inflammation may precede the onset of RA symptoms, also associated with ACPA [37]. Respiratory inflammation has also been described in ACPA positive individuals without RA [38]. Autoantibodies may also increase the risk of immune complex-mediated vascular inflammation [39]. Thus, CV disease and CV risk factors increase the future risk of RA development in the context of systemic inflammation and autoantibody development [12]. Patients with seropositive RA are at greater risk of CV disease [6,40], and it is possible that the RA genetic background is more permissive to atherosclerosis when associated with the “lifestyle” factors described here. In contrast to RA, in osteoarthritis inflammation is local and low-grade rather than systemic, and is not associated with autoantibodies.

**Figure 1:**
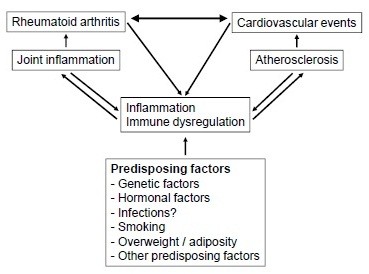
**Proposed model for illustrating the relationship among RA, CV disease, inflammation, atherosclerosis and predisposing factors.** CV, cardiovascular; RA, rheumatoid arthritis.

Figure 1 proposes a model for the relationships among RA, CV disease, inflammation and various predisposing factors, based on the results from the present study, as well as previous studies demonstrating an increased risk of CV disease after diagnosis of RA. Given that our findings were robust when adjusting for known risk factors for RA, this hypothesis merits further investigation in studies designed to clarify pathogenetic mechanisms, which is not possible in an epidemiological study.

## Conclusions

A history of previous CV disease was associated with increased risk of incident RA but not osteoarthritis. A probable explanation is that increased systemic inflammation may contribute to the parallel development of atherosclerosis and RA during the pre-clinical period in individuals exposed to common RA and CV risk factors, in a similar way that such interaction accelerates CV disease in patients with established RA. The association of previous CV events with the development of RA at the population level suggests presentation with CV events, especially in middle-aged female smokers or first-degree relatives of RA patients, should raise clinical suspicion to capture cases of undiagnosed early RA. Because CV risk factors are increased at the onset of RA, active cardiovascular risk management is important from the time of diagnosis.

## Abbreviations

ACPA: Anti-citrullinated peptide antibodies; BMI: Body mass index; CV: Cardiovascular; HDL: High-density lipoprotein; HUNT: Nord-Trøndelag Health Study; LDL: Low-density lipoprotein; MI: Myocardial infarction; OA: Osteoarthritis; RA: Rheumatoid arthritis.

## Competing interests

The authors declare that they have no competing interests.

## Authors’ contributions

HP was responsible for conception and design, data collection and analysis, and manuscript writing. MAB contributed to conception and design, data collection, and critical revision of the manuscript. SP contributed to conception and design, data analysis, and manuscript writing. RT took part in conception and design, and critical revision of the manuscript. VV was responsible for conception and design, data collection and analysis, and critical revision of the manuscript. All authors read and approved the final manuscript.
